# Infants differentially extract rules from language

**DOI:** 10.1038/s41598-021-99539-8

**Published:** 2021-10-08

**Authors:** Iris Berent, Irene de la Cruz-Pavía, Diane Brentari, Judit Gervain

**Affiliations:** 1 Northeastern University, Boston, MA USA; 2 Integrative Neuroscience and Cognition Center, Université de Paris & CNRS, Paris, France; 3 University of the Basque Country UPV/EHU, Vitoria-Gasteiz, Spain; 4 Basque Foundation for Science Ikerbasque, Bilbao, Spain; 5 University of Chicago, Chicago, USA; 6 University of Padua, Padua, Italy

**Keywords:** Human behaviour, Language

## Abstract

Infants readily extract linguistic rules from speech. Here, we ask whether this advantage extends to linguistic stimuli that do not rely on the spoken modality. To address this question, we first examine whether infants can differentially learn rules from linguistic signs. We show that, despite having no previous experience with a sign language, six-month-old infants can extract the reduplicative rule (AA) from dynamic linguistic signs, and the neural response to reduplicative linguistic signs differs from reduplicative visual controls, matched for the dynamic spatiotemporal properties of signs. We next demonstrate that the brain response for reduplicative signs is similar to the response to reduplicative speech stimuli. Rule learning, then, apparently depends on the linguistic status of the stimulus, not its sensory modality. These results suggest that infants are language-ready. They possess a powerful rule system that is differentially engaged by all linguistic stimuli, speech or sign.

## Introduction

Language allows humans to communicate novel messages reliably. People attain this feat because they are equipped with a powerful system of abstract rules^1–3^.

Past research has shown that speech—the most common channel of linguistic communication—is readily amenable to rule learning (e.g.,^4–20^). A large literature has focused on learning the reduplication rule (e.g., *pelala*, generally: ABB). Reduplication is of interest because it is prevalent across human languages^21–23^. For example, in Hawaiian, the word *hoe* ‘to paddle’ gives rise to *hoe-hoe* ‘to paddle continuously’^24^; Ilocano, a language spoken in the Philippines, uses reduplication to express plurality, as in *púsa* ‘cat’ and *puspúsa* ‘cats’^21^. Critically, young infants can freely extract the reduplication rule from speech^4–13^. In fact, humans can extract such tacit rules already at birth^13^.

Speech, however, is only one of the multiple modalities of human language^25–27^. A critical unanswered question is whether rule extraction is possible from a linguistic signal that is not spoken. Sign language offers a unique opportunity to address this question.

Manual signs are a natural form of human linguistic communication, and Deaf individuals are known to spontaneously generate sign languages anew^28–30^. Here, we ask whether young infants, who have no experience with any natural sign language, are able to extract rules from signs similarly to how they extract rules from speech. This allows us to evaluate whether human infants are prepared to extract linguistic rules across its multiple natural modalities.

Only two previous studies have examined whether infants can extract rules from signs^31,32^. The results were inconclusive. One study observed rule learning only for ABB, but not AAB sequences^31^. In the other study, signs failed to support rule learning altogether when the conversational setting was lacking^32^. This is in line with the results of a meta-analysis, where rule learning was most likely for meaningful stimuli^32^. The meta-analysis, however, mostly included studies on spoken language. The question thus arises whether hearing infants can extract rules from signs. Another critical question is whether infants can extract such rules *spontaneously*, even in the absence of an explicit conversational setting. Crucially, going beyond simply asking whether learning is possible, one important unknown issue is whether the mechanisms, as indexed by the neural systems, that support rule learning from signs overlap with the ones supporting rule learning from speech—no previous research has addressed this question.

In the current study, we used NIRS brain imaging to test 6-month-old infants’ ability to discriminate reduplication rules (AA) from non-reduplicative sequences (AB) implemented with dynamic signs (Exp 1) as well as with non-linguistic visual analogs (Exp 2). In both studies, we used a paradigm similar to the one employed for testing the extraction of reduplicative rules from speech^9,13^, allowing a direct comparison of the brain responses observed in the current study for signs and non-linguistic visual stimuli with those observed for speech. Our participants had no previous experience with sign languages or baby sign, and were thus not familiar with language in the visual-gestural modality.

**Figure 1:**
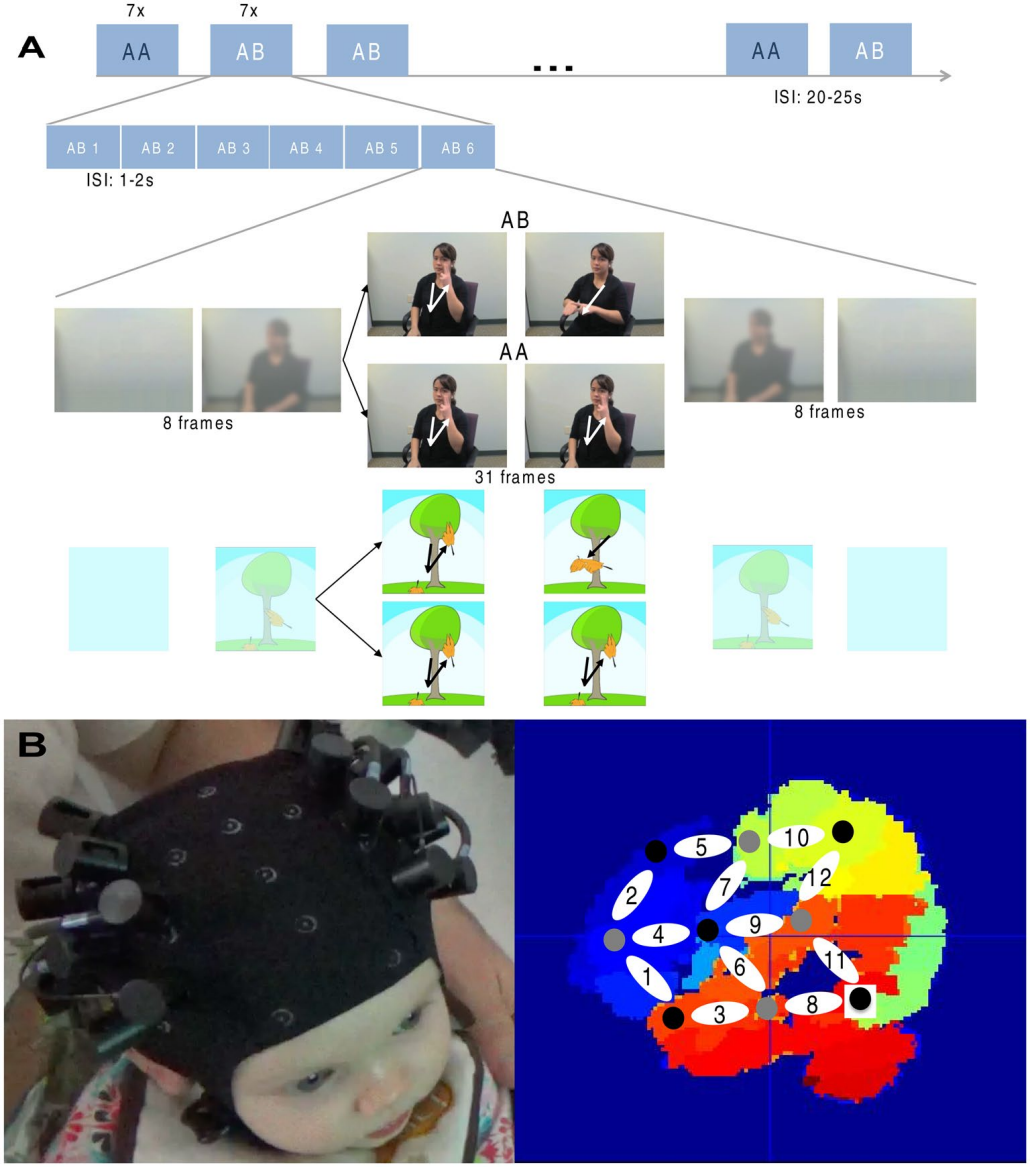
(**A**) The design and the stimuli of Experiments 1 and 2. Upper boxcar: Each experiment featured stimuli of two types: AA and AB. Each condition was presented in seven blocks for a total of 14 blocks. Each block featured six distinct stimulus pairs—either linguistic signs (Experiment 1) or non-sign visual analogs (Experiment 2). One example of an AB block is shown in the second boxcar plot. AA blocks had a similar structure. The AA and AB blocks were interspersed and their order was counterbalanced across participants. Lower rows: examples of a matched pair of AA and AB signs, and the corresponding non-sign analogs. Each such sign pair was comprised of 31 frames, preceded and followed by six faded frames and two additional frames. (**B**) left: a participant wearing the NIRS cap; right: localization of the NIRS channels: the optode configuration was overlaid on an average 6-month-old brain atlas; blue: frontal lobe, red: temporal lobe, yellow: parietal love, green: occipital lobe. The localization analysis following Lloyd-Fox et al.^33^ and Abboub et al.^34^, using age-appropriate structural MRIs and stereotaxic atlases^35,36^ suggests that channels 1, 2, 4, 5 and 13–16 query the frontal lobe, channels 3, 8, 11, 17, 22, and 24 are positioned over the temporal lobe, channels 10, 12, 20 and 23 are parietal, whereas channels 6, 7, 9, 18, 19 and 21 span two lobes.

Experiment 1 thus presented infants with dynamic linguistic signs of two types. One type was reduplicated (AA), with two identical syllables; another consisted of non-identical syllables (AB). Reduplicated and non-reduplicated signs were presented in separate, interleaved blocks in a within-subject design. Each block featured six signs (separated by blank screens), and each condition included a total of seven such blocks. The two conditions were presented in pseudorandomly interleaved blocks, the order of which was counterbalanced across participants (Fig. 1A). Experiment 2 compared infants’ brain responses to visual analogs of signs. These non-sign analogs were dynamic drawings of a tree, fitted with hand-shaped leaves that moved analogously to the human hands in Experiment 1. To match the sign to the nonsign analogs, we super-imposed the spatiotemporal properties of the signs (from Experiment 1) on the leaves (in Experiment 2) on a frame-by-frame basis. In so doing, we sought to precisely match the signs’ dynamic properties, and presented them in the same order as signs in Experiment 2.

Infants’ brain responses were measured using NIRS in the bilateral temporal, parietal and frontal regions (Fig. 1B), i.e. in the brain network known to be involved in language processing, both spoken^37^ and signed^38^ in adults, and spoken language processing in infants^9,39–41^. Specifically, these brain areas have been shown to support newborns’ capacity to learn reduplicative rules from speech in a NIRS paradigm similar to the one used here^9,13^.

If infants can extract rules from signs, then reduplicative signs (AA) should elicit different brain responses than non-reduplicative (AB) controls. If this rule system is differentially tuned to signs, then the effect of reduplication for signs (gauged by the AA/AB contrast) should differ from the responses to (nonlinguistic) visual analogs. Finally, if this differential learning from signs reflects tuning to language, then rule learning from signs should be similar to rule learning from speech.

## Results

**Figure 2:**
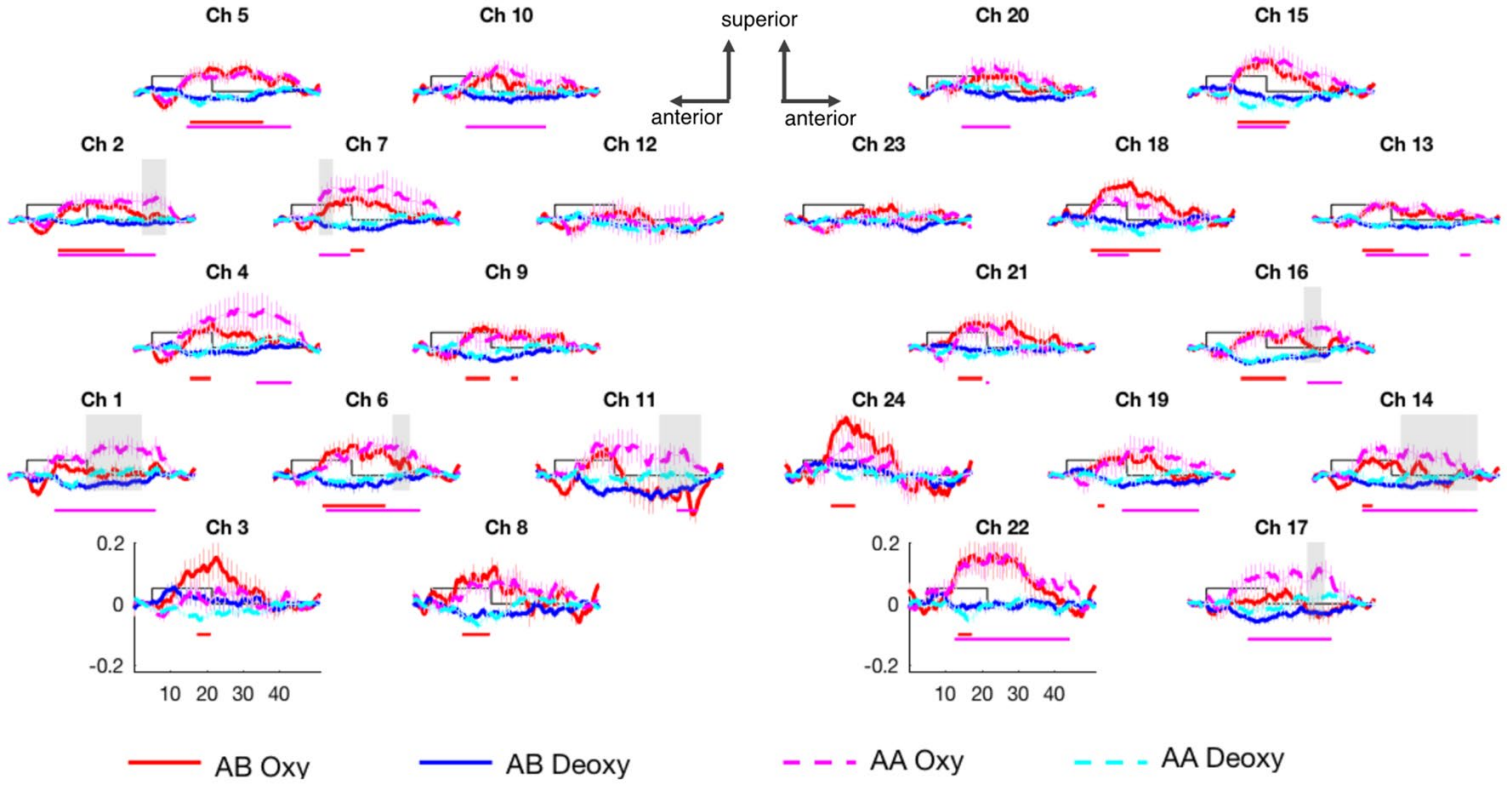
The grand average hemodynamic responses obtained in Experiment 1. Channels are plotted following the same placement shown in Fig. 1B. The x-axis represents time in seconds, the y-axis shows concentration in mmol*mm. The rectangle along the x-axis indicates time of stimulation in seconds. Continuous red and blue curves represent oxyHb and deoxyHb concentrations, respectively, in response to AB grammar. The dashed magenta and cyan curves represent oxyHb and deoxyHb concentrations, respectively, in response to AA signs. Lines placed below the plots indicate significant differences between the AB condition and baseline (red, top line) and the AA condition and baseline (magenta, lower line) as indicated by the permutation tests. For ease of exposition, only the time windows for oxyHb are shown. The results of the permutation tests for the direct comparison of the AA and AB conditions are indicated by gray-shaded rectangles overlaid on the curves. Error bars indicate standard errors of the means for every 20th data point in each curve.

**Figure 3:**
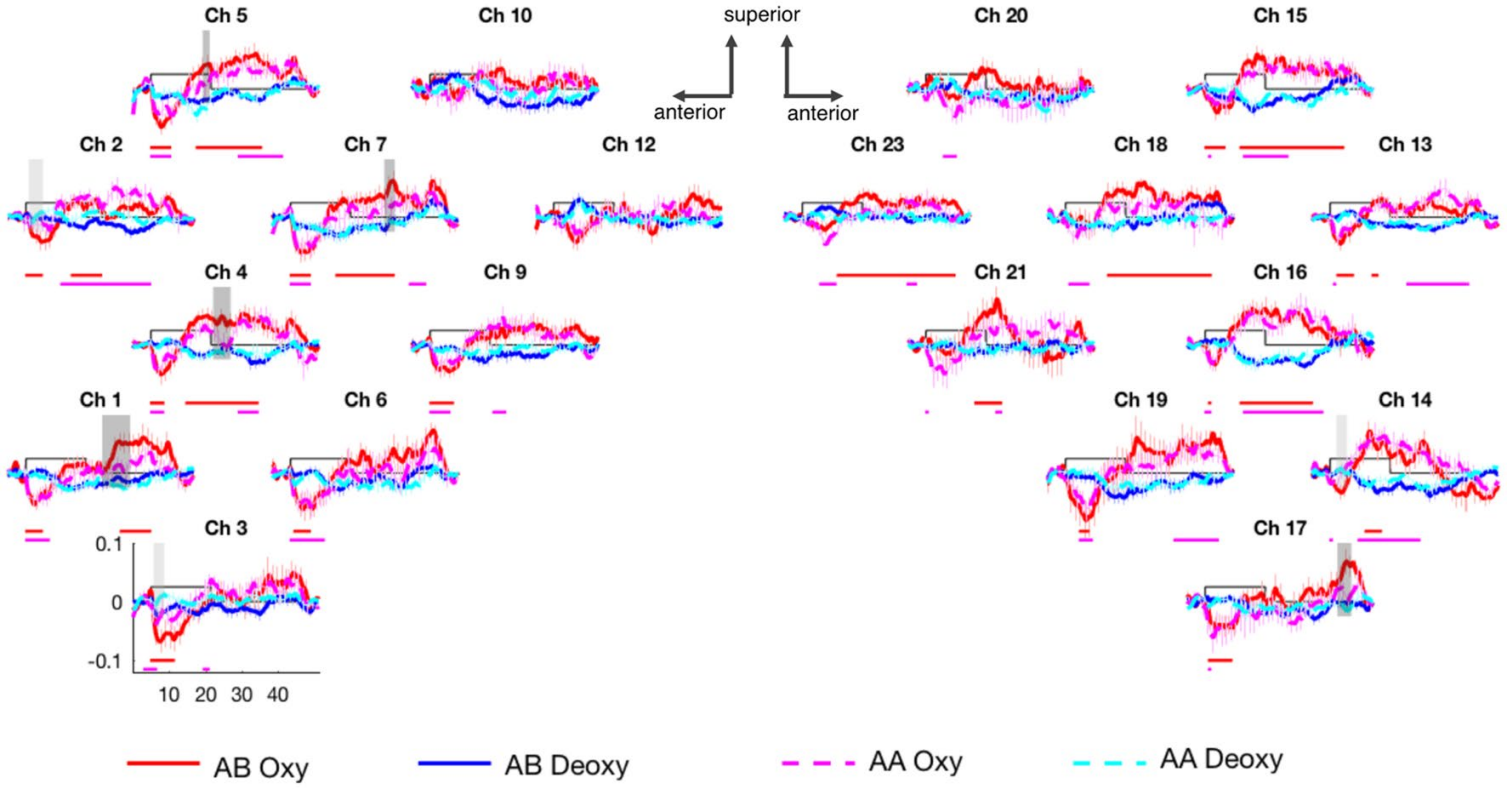
The grand average hemodynamic responses obtained in Experiment 2. Plotting conventions are the same as for Fig. 2 with the exception that the shaded areas showing significant differences in the permutation test between the two conditions are color coded to indicate separately differences occurring during the initial undershoot of the hemodynamic response (light gray) and the peak/plateau of the hemodynamic response (dark gray).

**Figure 4:**
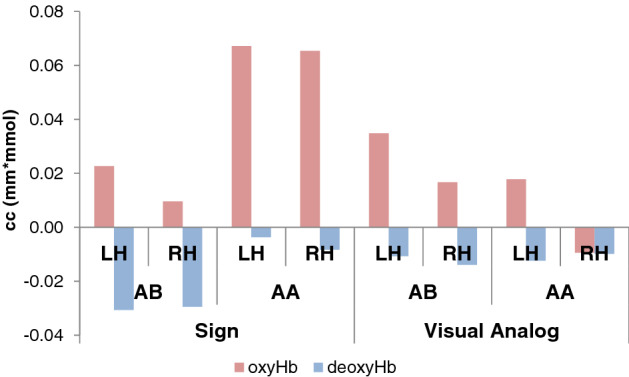
The grand average responses in Experiments 1 (Sign) and 2 (Visual Analog) in the spatial clusters identified by the permutation test.

Results showed that infants discriminated between the AA and AB patterns in both experiments. Remarkably, the effect of reduplication differed for linguistic signs and nonlinguistic visual controls. While signs (in Experiment 1) elicited greater activation for AA relative to AB sequences (Figs. 2 and 4) in the bilateral fronto-temporal areas, visual analogs (in Experiment 2) elicited the opposite pattern in the left fronto-temporal and the right temporal areas (Figs. 3 and 4). Thus, infants extracted rules from both signs and nonsigns, but the brain signatures of reduplication in linguistic and nonlinguistic stimuli diverged. For linguistic signs, reduplicative forms (AA) yielded greater activation than non-reduplicative forms; for nonlinguistic stimuli, activation was instead higher in response to non-reduplicative forms (AB).

In what follows, we first analyze the responses to signs and visual analogs separately. A subsequent set of analyses compares the responses to linguistic signs and visual analogs to each other, as well as to newborns’ responses to reduplication in speech obtained previously^9^.

### Responses to linguistic signs

A cluster-based permutation test comparing the responses to the AA and AB sequences with signs (in Experiment 1) revealed greater activation, as indexed by concentration changes of oxyhemoglobin (oxyHb), in response to the AA than to the AB sequences in the bilateral fronto-temporal areas, involving the superior temporal gyrus and the inferior temporal gyrus, including Broca’s region (Figs. 2 and 4).

Specifically, the permutation test directly comparing the AA and AB conditions showed significantly greater activation to AA than to AB sequences in channels 1, 2, 6, 7 and 11 in the LH and in channels 14, 16, 17 and 20 in the RH (all p < 0.001; the time windows of significance are indicated in Fig. 2). In the LH, channels 1, 6 and 11 formed a spatial cluster with a response significantly higher for AA than for AB (p < 0.001). In the RH, channels 14, 16 and 17 formed a spatial cluster (p < 0.001; Fig. 4). A permutation test directly comparing the AA and AB conditions using deoxyhemoglobin (deoxyHb) showed no significant differences, as is often the case in infant NIRS data^42^.

The permutation tests comparing each condition to baseline are reported in the Supplementary Material and the significant results are shown in Fig. 2.

### Responses to visual analogs

Unlike for linguistic signs, for the visual analogs (in Experiment 2), the permutation test for oxyHb showed greater peak responses to the AB than to the AA sequences in the left fronto-temporal and the right temporal regions (Figs. 3 and 4).

Specifically, the permutation test directly comparing the AA and AB conditions showed significantly greater activation to AB than to AA sequences in channels 1, 2, 3, 4, 5, and 7 in the LH and in channels 14, and 17 in the RH (all p < 0.001; the time windows of significance are indicated in Fig. 3). In the LH, channel 2 formed a spatial cluster in which the initial undershoot was significantly greater (more negative) in the AB than in the AA condition (p < 0.001), and channels 1, 4, 5, and 7 formed a spatial cluster in which the peak of the response was significantly higher for AB than for AA (p < 0.001). In the RH, channel 14 constituted a spatial cluster with a significant initial undershoot difference, and channel 17 formed a spatial cluster with a significant response peak difference (p < 0.001; Fig. 4). A permutation test directly comparing the AA and AB conditions using deoxyHb showed no significant results.

The permutation tests comparing each condition to baseline are reported in the Supplementary Material and the significant results are shown in Fig. 3.

### Comparing speech, linguistic signs, and visual analogs

The results described thus far show that reduplication elicited opposite responses in linguistic signs and nonsign visual controls. But is rule learning from signs similar to rule learning from the other channel of language—speech?

To address this question, we next compared the effect of reduplication in signs and speech—stimuli that are likewise linguistic, but that markedly differ from signs in their modality and surface characteristics. If the rule system is differentially tuned to all linguistic stimuli, then reduplication should elicit similar effects for speech and signs (which are both linguistic), but different from the effect of reduplication for nonlinguistic stimuli (nonsigns).

To test this, we compared the effect of reduplication in sign and in nonsign analogs to newborns’ responses to reduplication and no-reduplication rules in speech stimuli (data from Gervain et al.^9^, Experiment 1, contrasting ABB and ABC sequences). Newborns present an appropriate comparison because their lack of familiarity with natural speech approximates six-month-olds’ lack of familiarity with signs as closely as possible.

**Figure 5:**
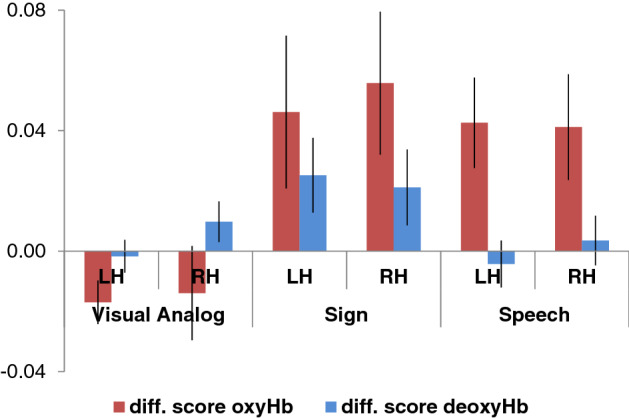
A direct comparison of the effect of reduplication (responses to reduplicative minus responses to non-reduplicative sequences) in the significant clusters in Experiments 1 & 2 in the current study (Exp 1: channels 1, 6, 11 in the LH & 14, 16, 17 in the RH; Exp 2: channels 1, 4, 5, 7 in the LH & 17 in the RH;) and in Exp 1 from Gervain et al.^9^ (a permutation test comparing ABB vs. ABC sequences revealed a significant cluster including channels 3, 4, 6 and 9 in the LH and channels 16 and 19 in the RH). Errors bars represent standard errors of the mean.

For this comparison, we obtained oxyHb and deoxyHb concentration changes in the significant clusters in each hemisphere in Experiments 1–2 of the current study as well as in Experiment 1 from Gervain et al. (2008). The concentrations were normalized to the baseline, as is necessary for between-subject comparisons with NIRS data^34,43,44^. To evaluate the effect of reduplication, we calculated a difference score (for both oxyHb and deoxyHb), by subtracting the responses to the non-reduplicative (AB/ABC) sequences from the responses to the reduplicative (AA/ABB) sequences (Fig. 5). This controls for overall baseline differences between Experiments 1–2 and the Gervain et al.’s previous study with respect to participants’ age, stimulus length, and the settings of the NIRS methods. A similar analysis, conducted over the oxyHb and deoxyHb concentrations (rather than over the difference scores) yielded comparable results (reported in the Supplementary Materials). In the present analysis, difference scores close to zero indicate no difference between the two structures, whereas positive scores suggest that reduplication elicited a stronger hemodynamic response.

We conducted an ANOVA with Stimulus Type (Sign/Visual Analog/Speech) as a between-subjects factor and Hemisphere (LH/RH) as within-subjects factors over the difference scores for oxyHb. We obtained a main effect of Stimulus Type (F(2, 63) = 4.5223, p = 0.014). The main effect of Hemisphere was not significant (F(1,61) < 1), nor was the Stimulus Type x Hemisphere interaction (F(2,61) < 1). LSD post hoc tests indicated that this main effect is due to a positive effect of reduplication in the Sign condition as compared to the Visual Analog condition, where the effect of reduplication was negative, i.e. activation was greater for the non-reduplicated sequences (p = 0.007, Cohen’s *d* = 0.342). The effect of reduplication was also greater in the Speech condition than in the Visual Analog condition (p = 0.017, Cohen’s *d* = 0.291). Remarkably, the effect of reduplication for Signs did not differ from the Speech condition (p = 0.725, n.s.). To follow up on these results and test whether this non-significant difference provides support for the null hypothesis that Speech and Sign are actually similar, and to assess how strongly the data supports the difference between sign and speech on the one hand and the visual analog on the other hand, we ran a Bayesian version of the same ANOVA. The most probable model was the one with a main effect of Stimulus Type. This model was substantially more likely than the null model (subject as a random factor only) with BF_10_ = 3.500. The other three models (main effect of Hemisphere, main effects of Hemisphere and of Stimulus Type and the full model) were less likely than the null model given the data. Most importantly, the post hoc comparisons for the main effect of Stimulus Type yield substantial evidence for the null hypothesis that Speech and Sign are similar (BF_10_ = 0.246) as well as strong evidence for the difference between Signs and Visual Analogs (BF_10_ = 19.792) and decisive evidence for the difference between Speech and Visual Analogs (BF_10_ = 115.331) A similar analysis over deoxyHb difference scores yielded no significant results.

Thus, infants responded *differently* to the reduplication rule in linguistic signs and nonlinguistic controls that were closely matched for their sensory demands, but they responded *similarly* to linguistic stimuli that vastly contrasted on their sensory characteristics—spoken (auditory) and signed (visual). Sensitivity to rules, then, was unaffected by the sensory modality of the stimulus, but it was selectively tuned to its linguistic status.

## General discussion

Human language allows us to produce and comprehend sentences that we have never heard before. This quintessential human capacity arises because human language relies on powerful rules^1,45^. Past research has shown that human infants can readily extract rules from speech stimuli^4–13^ and they do so already at birth^13^. But whether this early system of rule learning is tuned to language generally or only to speech is unknown. Since infants are exposed to speech already in utero^46^, it is conceivable that speech is amenable to rule learning because it is familiar, not because it is a carrier of language.

To address this issue, this research asked two questions. First, are infants differentially tuned to linguistic rules in signs (as compared to nonlinguistic visual stimuli)—stimuli that are likewise linguistic, but unfamiliar to infants? Second, is rule learning from signs similar to rule learning from speech?

Our results showed that, despite having no previous experience with a sign language, infants spontaneously extracted the reduplication rule from linguistic signs, while reduplication in nonlinguistic controls elicited a markedly different pattern. In particular, we found that in signs, reduplication elicited higher activation relative to controls, whereas for sign analogs, reduplication yielded lower activation relative to controls. Importantly, we did not simply measure responses to sign and non-sign stimuli. Rather, it is the effect of the reduplication *rule* (i.e., ∆_ABB-ABC_ and ∆_AA-AB_) that differed in these two experiments. These results suggest that infants can extract rules from linguistic signs, and the pattern of results differs from infants’ brain responses to rules in closely matched nonlinguistic stimuli.

We next asked whether the differential response to rules in signs are similar to rule learning from speech. We found that while infants’ response to reduplication in signs differed from nonlinguistic visual controls, it was statistically indistinguishable from their response to reduplication in speech (^9^). Moreover, the brain networks involved were similar, as is also the case in the adult brain, which processes spoken and signed languages involving a predominantly left-lateralized perisylvian network^38^.

Altogether, then, our results demonstrate for the first time a *double dissociation* between the sensory modality of linguistic stimuli and their linguistic status. Linguistic signs and nonsign controls shared the visual modality and they were strictly matched for their spatiotemporal characteristic, yet responses to reduplication in these stimuli differed markedly. In contrast, signed and spoken stimuli rely on different sensory modalities, but they are both linguistic. Despite their sensory differences, speech and signs were equally amenable to the extraction of reduplication. As such, these results suggest that infants possess an amodal system of rule learning that is differentially tuned to linguistic rules in all forms of language—speech and sign.

Why are speech and signs preferentially targeted by the rule-learning system? Our present results cannot fully answer this question. One possibility is that this tuning arose because the rule learning system is *inherently* tuned to language, and speech and signs are its natural channels (hereafter, the *linguistic* hypothesis).

Alternatively, the advantage of speech and signs may not be inherent to language per se. As noted, infants might be tuned to speech because it is familiar—they are exposed to speech already in utero, albeit in a strongly filtered form^46^. The preference for signs could have likewise arisen because signs feature human agents, because infants are exposed to co-speech gestures, so they consider the signers’ hand gestures as signaling communication, or because these stimuli are particularly “meaningful” to infants. According to this alternative explanation, speech and signs are not inherently different from nonlinguistic stimuli. The similarity in response between signs and speech only arises because these stimuli are associated with certain correlates (e.g., familiarity, human gestures, meaningfulness), and these correlates happen to elicit similar effects on the rule learning system (hereafter, the *nonlinguistic* hypothesis).

While our study cannot definitively rule out the possibility that the differential tuning of the system to speech and signs arises for reasons that are not inherently related to their roles as carriers of language, we note several challenges to this nonlinguistic hypothesis.

One challenge is to explain the *differential* responses to signs (relative to the visual controls). The fact that signs feature human agents (complete with faces and hands), and that signing could suggest a communicative intent could both explain the sign advantage. Past research, however, found no consistent rule learning from the manual gestures of human agents^31,32^, suggesting that the presence of a human agent (including its face and hands) or unidirectional communicative intent is insufficient to account for the extraction of rules we had observed in signs (nor can it capture the opposite effect of reduplication in nonsigns). Similar challenges face the hypothesis that linguistic signs are preferred to nonsigns because the human agent (in signs) is “meaningful”^32^. The first problem is how “meaningfulness” may be best defined. One possibility is that meaningful stimuli are ones that can be linked to a referent. Since our control stimuli (depicting dynamic tree-like cartoons) do meet this condition, and since past research shows that infants can recognize dynamic cartoons^47^, we would expect them to encode the tree-like nonsign cartoons as meaningful, akin to the human signs. “Meaningfulness”, then, should predict similar responses to signs and nonsigns. These predictions are inconsistent with our results.

Another challenge to the nonlinguistic hypothesis arises from the *similar* effect of reduplication for speech and sign, despite marked differences in their sensorimotor properties. Also, our analysis controls for general differences in the processing of the three different types of stimuli, as we are specifically testing the effect of reduplication (∆_ABB-ABC_ and ∆_AA-AB_) and not the general response to the nature of the stimuli.

Beyond these empirical challenges, the nonlinguistic hypothesis also faces a conceptual problem. The challenge is to explain why the disparate sensorimotor features of speech and signs happen to converge. That is, why features such as the dynamics of the human hand or the face and the formant structure of speech sounds are treated similarly by human infants. Why do these distinct attributes trigger the same rule learning response in the infant brain? The hypothesis that these stimuli are treated alike *because* they are potential carriers of language offers a simple explanation to this puzzle. Beyond its ability to capture the empirical findings, this proposal further offers a unified framework to account for the phylogenetic and ontogenetic emergence of co-speech gestures and conventionalized, semantically meaningful gestures accompanying speech. In this view, it is not sign language that piggybacks on co-speech gestures, but rather the other way around. Co-speech gestures arise because human brains are tuned to *language*, aural and manual, and co-speech gestures exploits this readiness.

But if the similarities between the neural processing of signs and speech indeed arise from their status as carries of language, then how do infants recognize linguistic stimuli as such? What properties of signs, for example, designate them as “linguistic” stimuli, akin to speech, but distinct from visual objects? While our experiments are not designed to decide this issue, several possibilities come to mind. One is that linguistic stimuli are identified by attending to phonetic cues, such as the formant structure and articulatory gestures of speech^48^, or the specific rhythmic periodicity of hand movements^49^. Another possibility is that linguistic input is identified by virtue of their structure: well-formed structures might be more likely to be identified as potentially “linguistic”, and thus, amenable to rule-learning. This hypothesis explains why infants in our experiments were readily able to extract rules from the AA signs (as these structures are frequent in sign languages, hence, potentially well-formed^22,23^), whereas past research^31,32^ failed to find consistent rule learning from ABB/AAB forms (which are relatively rare, hence, possibly ill-formed) (Trisyllabic AAB and ABB signs each account for less than 1% of the signs in ASL^50^, and as such, they are potentially ill-formed.). Finally, linguistic stimuli might also be identified as such by tracking the speakers’ communicative intent^32,51^. Given that our sign stimuli were strictly matched to nonsigns with respect to periodicity, and they did not explicitly reveal communicative intent between interacting social agents, it is unlikely that any of these factors singlehandedly defines language as such. Nonetheless, it is conceivable that phonetic cues (e.g., hand movement, shape and location, for sign language), linguistic structure, and pragmatics could all combine synergistically to render novel signs more accessible to linguistic computations.

How linguistic stimuli are identified as such, and why they are particularly amenable to the extraction of rules are important questions that the present study leaves open for future research. These limitations notwithstanding, our results do show for the first time that rules are preferentially extracted from linguistic stimuli and this is achieved by similar brain mechanisms even in young infants, irrespective of the modality.

Linguistic rules are at the core of the human capacity for language^2,45^, and they are expressed in two distinct modalities—speech and sign. Our findings suggest that the human capacity to learn rules is supported by a brain system that is narrowly tuned for language but broad with respect to its input modality. This shared amodal system is further evident already within the first months of life. Consequently, human infants are not only speech-ready; they are language-ready. They possess the computational means to extract linguistic structure from natural language in all forms—aural or manual.

## Materials and methods

### Participants

#### Experiment 1

Twenty-three healthy French-learning infants (12 females; mean age: 5 months 27 days; range: 5 months 14 days–6 months 6 days) contributed to the final analyses. No participants were exposed to sign language, or “baby sign”. Forty-nine additional infants were tested, but not included in the data analysis due to fussiness and crying (28), an insufficient number of valid trials/poor data quality (20) or parental interference (1). Rejection due to poor data quality was performed in batch, following the same criteria for all infants (see Data Processing and Analysis), prior to statistical analysis. All parents gave written informed consent before the experiment. The study was approved by the CERES ethics board (Université de Paris). We obtained written informed consent from the signer (in Fig. 1a) and from the parents of the infant (in Fig. 1b) to publish their respective images in open-access publication.

### Experiment 2

Twenty-one healthy French-learning infants (7 females; mean age: 5 months 29 days; range: 5 months 17 days–6 months 15 days) contributed data to the final analyses. Forty-three additional infants were tested, but were not included in the data analysis due to fussiness and crying (28), an insufficient number of valid trials/poor data quality (13) or parental interference (3). Rejection due to poor data quality was performed in batch, following the same criteria for all infants (see Data Processing and Analysis), prior to statistical analysis. All parents gave informed consent before the experiment. The present experiment was approved by the CERES ethics board (Université de Paris). All methods were performed in accordance with the relevant guidelines and regulations.

### Design and stimuli

#### Design

Experiment 1 featured 42 pairs of dynamic disyllabic signs, matching one reduplicated sign (AA) with a non-reduplicated control (AB). Each disyllabic sign lasted 47 frames (1.6 s). Reduplicated and non-reduplicated signs were presented in separate blocks, in seven blocks per condition for a total of 14 blocks. Each block consisted of 6 different disyllabic signs, separated by pauses (grey screen) varying between 1–2 s. AA and AB blocks were strictly matched for the duration and order of pauses as well as for the ordering of the specific A and B syllable tokens within a block. Each block lasted a total of approximately 16.6 s. The order of blocks was counterbalanced across participants in a Latin Square design. Blocks were separated by baseline videos of 20–25 s consisting of non-object-like moving rays of light changing color. The whole experiment lasted about 9 min (for additional details, see SM).

Experiment 2 featured visual analogs of the signs used in Experiment 1. They were derived from the signs in the following way*.* A trained video artist constructed the analogs from a still drawing of a tree and two leaves. The tree trunk and tree top were drawn to roughly match the contour of the signer’s torso and head, whereas the shape of each of the leaves and their position were matched to approximate the five fingers of the signer’s hand. These animations were next edited to match the signer’s hand position and movement for each sign on a frame-by-frame basis. Accordingly, the orientation of the leaves and their position relative to the tree trunk were strictly matched to the orientation of the signer’s hands and their position relative to her body. Additionally, for each sign, the tree top was further centered and aligned with the signer’s head, and the tree top moved to match the trajectory of the head on a frame-by-frame basis (for additional details, see SM).

### Procedure

Infants in Experiment 1 were tested with a NIRx NIRScout 16–16 machine (source-detector separation: 3 cm; two wavelengths of 760 nm and 850 nm; sampling rate: 15.625 Hz) at the maternity unit of the Robert Debré Hospital, Paris, France (n = 13) or with a NIRx NIRScout 8–16 machine (same characteristics as the NIRScout 16–16) in a quiet, dimly lit testing booth at the Integrative Neuroscience and Cognition Center, Université de Paris (n = 10). The optical sensors were inserted into a stretchy cap and placed on the infants’ head using surface landmarks (nasion, and the preauricular points), targeting the language areas in the bilateral temporal, frontal, and parietal cortices. These areas match those that responded to reduplication in speech in newborns (Gervain et al. 2008). While we used visual stimuli, we did not measure from the occipital areas, as the visual processing of the stimuli was not relevant for our purposes. We approximated the cortical regions underlying our NIRS channels following Lloyd-Fox et al.^33^ and Abboub et al.^34^, using age-appropriate structural MRIs and stereotaxic atlases^35,36^. The position of optodes was measured with respect to the nasion and tragi for each participant and, together with photographs of the optode positions, were used to localize the optodes on a structural whole head MRI image. The locations were then projected down onto the cortical surface to identify the regions underlying the NIRS channels for each infant. A channel was then labeled according to the localization found in the majority of participants. Accordingly, channels 1, 2, 4, 5 and 13–16 query the frontal lobe, channels 3, 8, 11, 17, 22, and 24 are positioned over the temporal lobe, channels 10, 12, 20 and 23 are parietal, whereas channels 6, 7, 9, 18, 19 and 21 span two lobes.

During testing, infants were seated on a caregiver’s lap in a quiet, dimmed room. The stimuli were presented on a screen in front of the infants at approximately 80 cm using E-Prime. If infants looked away or lost attention during the baseline periods, an experimenter hidden behind the computer screen presented silent toys to redirect infants’ attention.

Experiment 2 used the same general procedure with two exceptions. Participants were tested in a quiet, dimly lit testing booth at the Integrative Neuroscience and Cognition Center, Université de Paris. The setup was similar to the one used in Experiment 1, but there was no space behind the screen for the experimenter to hide, so no silent toys were used to reorient infants’ attention. Also, the NIRS machine used was a NIRx NIRScout 8–16, which had only 8 and not 16 detectors. As a result, only 20 and not 24 channels were available, resulting in a configuration which was similar to the one used in Experiment 1, but lacked channels 8 and 11 in the LH and channels 22 and 24 in the RH.

### Data processing and analysis

The NIRS machine measured the intensity of the transmitted light, from which concentration changes of oxygenated hemoglobin (oxyHb) and deoxygenated hemoglobin (deoxyHb) were calculated using the modified Beer-Lambert Law. To eliminate noise (e.g., heartbeat) and overall trends, the data were band pass-filtered between 0.01–0.7 Hz. Movement artifacts, defined as concentration changes above 0.1 mmol*mm over two samples, were removed by rejecting block-channel pairs in which artifacts occurred. For valid, non-rejected blocks, a baseline was linearly fitted between the means of the 5 s preceding the onset of the block and the 5 s starting 15 s after the offset of the block. Infants were videotaped during the experiment. Videos were coded offline and blocks during which the infant did not watch at least one disyllabic sign were rejected in all channels. Infants were included in the analysis if they had at least 33% valid data. In Experiment 1, the 23 infants included in the final sample provided 55% valid data after data quality and looking behavior were taken into account. In Experiment 2, the 21 infants included in the final sample provided 74% valid data once both data quality and looking behavior was taken into account.

Statistical analyses were carried out over both oxyHb and deoxyHb. We conducted cluster-based permutation analyses (Maris & Oostenveld, 2007) comparing each condition to a zero baseline as well as the two conditions between them. This identified spatially adjacent channels in which significant activation was observed in temporally adjacent samples. Permutation tests avoid the multiple comparison problem, and identify regions and time windows of interest in a non-arbitrary, data-driven fashion. To perform the permutation test, we used paired-sample t-tests with t = 2 as threshold for significance. We ran 1000 permutations under the null hypothesis. Additionally, we conducted an analysis of variance (ANOVA) to directly compare the two experiments to each other, as well as with newborns’ responses to reduplication (ABB) and no-reduplication (ABC) in speech (data from Gervain et al. (16)). The ANOVA comprised of the between subjects-factor Stimulus Type (Sign/Visual Analog/Speech) and the within-subject factor Hemisphere (LH/RH) of the difference score between the responses to reduplicated and non-reduplicated stimuli, normalized to their zero baselines, as is appropriate for between-subject NIRS comparisons, in the significant clusters revealed by the permutation tests (a permutation test was also conducted for the newborn data from^9^). Additionally, a similar ANOVA was run directly on concentration changes and is reported in the SM. Furthermore, to better assess non only the differences, but also the predicted similarities between the conditions, specifically speech and sign, we have conducted a Bayesian version of the ANOVA with the between subjects-factor Stimulus Type (Sign/Visual Analog/Speech) and the within-subject factor Hemisphere (LH/RH).

## Supplementary Information

Supplementary Information.
