# Supplementary material for: Infants differentially extract rules from language
Source: Sci Rep. 2021 Oct 8;11:20001. doi: 10.1038/s41598-021-99539-8 (PMC8501030; doi:10.1038/s41598-021-99539-8)
Supplement: Supplementary file 1 — Supplementary Information. [file 41598_2021_99539_MOESM1_ESM.docx]

**Infants differentially extract rules from language**

***Supplementary Materials***

Iris Berent^1*^, Irene de la Cruz-Pavía^2,3,4^, Diane Brentari^5^, Judit Gervain^2,6^

^1^ Northeastern University, Boston, MA, USA

^2^ Integrative Neuroscience and Cognition Center, Université de Paris & CNRS, Paris, France

^3^ University of the Basque Country UPV/EHU, Vitoria-Gasteiz, Spain

^4^ Basque Foundation for Science Ikerbasque, Bilbao, Spain

^5^ University of Chicago, USA

^6^ University of Padua, Padua, Italy

*Corresponding author

i.berent@northeastern.edu

**Experiment 1: Sign Language**

**Materials and Methods**

*Participants*

Twenty-three healthy French-learning infants (12 females; mean age: 5 months 27 days; range: 5 months 14 days – 6 months 6 days) contributed to the final analyses. No participants were exposed to sign language, or “baby sign”. Forty-nine additional infants were tested, but not included in the data analysis due to fussiness and crying (28), an insufficient number of valid trials / poor data quality (20) or parental interference (1). Rejection due to poor data quality was performed in batch, following the same criteria for all infants (see Data Processing and Analysis), prior to statistical analysis. All parents gave informed consent before the experiment. The study was approved by the CERES ethics board (Université de Paris).

*Design and Stimuli*

*Experimental Stimuli.* The stimuli were novel (i.e. non-existent) ASL signs, but were phonotactically legal, as verified by a phonologically trained native ASL signer. Signs were comprised of 21 quartets (e.g., x_1_x_1_, y_1_y_1_, x_1_y_1_, and y_1_y_1_, where x_1_ and y_1_ represent distinct syllables), such that any particular x and y syllables were equally likely to appear at the initial and final position of the sign. Within a quartet, the x and y syllables contrasted on most of their phonological features (their location, handshape and palm orientation). The signs were produced by a female native signer of ASL to the rhythm of a visual metronome (144 BPM rate) such that each disyllabic sign lasted exactly 31 frames (about 1.04 seconds). To smooth the appearance of the signs, we then added eight frames at the beginning and end of each recording, such that the initial and final six frames faded in or out, for a total of 47 frames.

The edited disyllabic signs were next combined to form the AA and AB blocks. Each block consisted of six disyllabic signs, separated by silences whose duration ranged from 1-2 seconds, measured from the offset of the original (unedited) sign to the onset of the next one. Block structure was counterbalanced using a Latin Square design to control for and match the occurrence of specific syllables in the initial and final position, as well as for the duration and order of intermediate silences. A sample of stimulus is available at url

<https://www.youtube.com/playlist?list=PLBdp4mOe9SrffjfvzZfNWIObFRasL4WSY>.

*Procedure*

Infants were tested with a NIRx NIRScout 16-16 machine (source-detector separation: 3 cm; two wavelengths of 760 nm and 850 nm; sampling rate: 15.625 Hz) at the maternity of the Robert Debré Hospital, Paris, France (n=13) or with a NIRx NIRScout 8-16 machine (same characteristic as the NIRScout 16-16) in a quiet, dimly lit testing booth at the Integrative Neuroscience and Cognition Center, Université de Paris (n=10). The optical sensors were inserted into a stretchy cap and placed on the infants’ head using surface landmarks (nasion, and the preauricular points), targeting the language areas in the bilateral temporal, frontal and parietal cortices. These areas match those that responded to reduplication is speech in newborns (Gervain et al., 2008). While we used visual stimuli, we did not measure from the occipital areas, as the visual processing of the stimuli was not relevant for our purposes. We approximated the cortical regions underlying our NIRS channels following Lloyd-Fox et al. (*1*) and (*2*), using age-appropriate structural MRIs and stereotaxic atlases ((*3, 4*)). The position of optodes was measured with respect to the nasion and tragi for each participant and, together with photographs of the optode positions, were used to localize the optodes on a structural whole head MRI image. The locations were then projected down onto the cortical surface to identify the regions underlying the NIRS channels for each infant. A channel was then labeled according to the localization found in the majority of participants. Accordingly, channels 1, 2, 4, 5 and 13-16 query the frontal lobe, channels 3, 8, 11, 17, 22, and 24 are positioned over the temporal lobe, channels 10, 12, 20 and 23 are parietal, whereas channels 6, 7, 9, 18, 19 and 21 span two lobes.

During testing, infants were seated on a caregiver’s lap in a quiet, dimmed room. The stimuli were presented on a screen in front of the infants at approximately 80cm using E-Prime. If infants looked away or lost attention during the baseline periods, an experimenter hidden behind the computer screen presented silent toys to redirect infants’ attention.

*Data Processing and Analysis*

The NIRS machine measured the intensity of the transmitted light, from which concentration changes of oxygenated hemoglobin (oxyHb) and deoxygenated hemoglobin (deoxyHb) were calculated using the modified Beer-Lambert Law. To eliminate noise (e.g., heartbeat) and overall trends, the data were band pass-filtered between 0.01-0.7Hz. Movement artifacts, defined as concentration changes above 0.1 mmol*mm over two samples, were removed by rejecting block-channel pairs in which artifacts occurred. For valid, non-rejected blocks, a baseline was linearly fitted between the means of the 5 s preceding the onset of the block and the 5 s starting 15 s after offset of the block. Infants were videotaped during the experiment. Videos were coded offline and blocks during which the infant did not watch at least one disyllabic sign were rejected in all channels. Infants were included in the analysis if they had at least 33% valid data. The 23 infants included in the final sample provided 55% valid data after data quality and looking behavior were taken into account.

Statistical analyses were carried out over both oxyHb and deoxyHb. We conducted cluster-based permutation analyses (Maris & Oostenveld, 2007) comparing each condition to a zero baseline as well as the two conditions between them. This identified spatially adjacent channels in which significant activation was observed in temporally adjacent samples. Permutation tests avoid the multiple comparison problem, and identify regions and time windows of interest in a non-arbitrary, data-driven fashion. To perform the permutation test, we used paired-sample t-tests with t = 2 as threshold for significance. We ran 1000 permutations under the null hypothesis.

**Results**

*Permutation tests*

*OxyHb.* The permutation test comparing the AB condition to baseline using oxyHb revealed a significant response in channels 2, 3, 4, 5, 6, 7, 8 and 9 in the LH and in channels 13, 14, 15, 16, 18, 19, 21, 22 and 24 in RH (p<0.001). Of these, the LH channels 2, 4, 5, 7 and 9 formed a spatial cluster in which the response was significantly different from baseline (p<0.001). In the RH, channels 13, 15, 16, 18 and 21 formed a spatial cluster with a significant response (p<0.001). The permutation test comparing the AA condition to baseline revealed significant responses in channels 1, 2, 4, 5, 6, 7 and 10 in the LH and channels 13, 14, 15, 16, 17, 18, 19, 20 and 22 in the RH (all p<0.001). In the LH, channels 1, 2, 4, 6 and 7 formed a spatial cluster with a significant response (p<0.001). In the RH, channels 14, 16, 17, 19 and 22 formed a spatial cluster with a significant response (p<0.001). The permutation test directly comparing the AA and AB conditions showed significantly greater activation to AA than to AB sequences in channels 1, 2, 6, 7 and 11 in the LH and in channels 14, 16, 17 and 20 in the RH (all p<0.001). In the LH, channels 1, 6 and 11 formed a spatial cluster with a response significantly higher for AA than for AB (p<0.001). In the RH, channels 14, 16 and 17 formed a spatial cluster (p<0.001).

*DeoxyHb.* The permutation test comparing the AB condition to baseline using deoxyHb revealed a significant decrease in channels 1, 7, 8, 9, 11 and 12 in the LH and in channels 14, 16, 17 and 19 in the RH (p<0.001). Of these, the LH channels 7, 9, 11 and 12, and the RH channels 14, 16, 17 and 19 formed spatial clusters (p<0.001). The permutation test comparing the AA condition to baseline revealed significant responses in channels 1, 3, 5 and 8 in the LH, and in channels 13, 15, 16 and 18 in the RH (p<0.001). Of these, the LH channels 1, 3, and 8 and the RH channels 15, 16 and 18 formed a spatial cluster (p<0.001). A permutation test directly comparing the AA and AB conditions showed no significant differences.

**Experiment 2: Visual Analogue**

**Materials and Methods**

*Participants*

Twenty-one healthy French-learning infants (7 females; mean age: 5 months 29 days; range: 5 months 17 days – 6 months 15 days) contributed data to the final analyses. Forty-three additional infants were tested, but were not included in the data analysis due to fussiness and crying (28), an insufficient number of valid trials / poor data quality (13) or parental interference (3). Rejection due to poor data quality was performed in batch, following the same criteria for all infants, prior to statistical analysis. All parents gave informed consent before the experiment. The present experiment was approved by the CERES ethics board (Université de Paris).

*Design and Stimuli*

The stimuli used in this experiment were visual analogues of the signs used in Experiment 1. Each nonsign was next edited, adding eight frames on each end (to match the edited signs). The ISI between consigns was calculated from the offset of one edited nonsign to the onset of the next. Nonsigns were next combined to form blocks, arranged to precisely match those of their sign equivalents. A sample stimuli is available at url [https://www.youtube.com/playlist?list=PLBdp4mOe9SrffjfvzZfNWIObFRasL4WSY](https://nam05.safelinks.protection.outlook.com/?url=https%3A%2F%2Fwww.youtube.com%2Fplaylist%3Flist%3DPLBdp4mOe9SrffjfvzZfNWIObFRasL4WSY&data=02%7C01%7CI.Berent%40northeastern.edu%7Cb5475866886c476f099208d6fb12534d%7Ca8eec281aaa34daeac9b9a398b9215e7%7C0%7C0%7C636972453470437942&sdata=%2BErmueNjSxWyHNKHPmmYiWfcu7o0ZukT1WLDpARPsc0%3D&reserved=0).

The baseline stimuli and the experimental design were identical to those used in Experiment 1.

*Procedure*

The general procedure was identical to Experiment 1, with two exceptions. Participants were tested in a quiet, dimly lit testing booth at the Integrative Neuroscience and Cognition Center, Université de Paris. The setup was similar to the one used in Experiment 1, but there was no space behind the screen for the experimenter to hide, so no silent toys were used to reorient infants’ attention. Also, the NIRS machine used was a NIRx NIRScout 8-16, which had only 8 and not 16 detectors. As a result, only 20 and not 24 channels were available, resulting in a configuration which was similar to the one used in Experiment 1, but lacked channels 8 and 11 in the LH and channels 22 and 24 in the RH.

*Data Processing and Analysis*

The data was preprocessed and analyzed in the same way as for Experiment 1. The 21 infants included in the final sample provided 74% valid data once both data quality and looking behavior was taken into account.

We performed the same permutation tests as for Experiment 1. Additionally, we conducted an analysis of variance (ANOVA) to directly compare the two experiments with between subjects-factor Stimulus Type (Sign/Visual Analog) and within-subject factors Structure (AA/AB) and Hemisphere (LH/RH) comparing the z-score transforms of the oxyHb concentration change, as is appropriate for between-subject NIRS comparisons, for the AA and AB conditions in the bilateral temporal channels 1 (LH) and 17 (RH), where activation overlapped between the two experiments as identified by the permutation test.

**Results**

*Permutation tests*

*OxyHb.* The permutation test comparing the AB condition to baseline using oxyHb revealed a significant response in channels 1, 2, 3, 4, 5, 6, 7, and 9 in the LH and in channels 13, 14, 15, 16, 17, 18, 19, 21 and 23 in RH (all p<0.001). Of these significant channels, the LH channels 1, 2, 3, 4, 6, 7 and 9 formed a spatial cluster in which the initial undershoot was significantly different from baseline (p<0.001), and channels 2, 4, 5 and 7 formed a spatial cluster in which the peak of the response was significantly different from baseline (p<0.001). In the RH, channels 13, 14, 15, 16, 17 and 19 formed a spatial cluster with a significant initial undershoot (p<0.001), and channels 15, 16, 18, 21 and 23 formed a spatial cluster with a significant response peak (p<0.001). The permutation test comparing the AA condition to baseline revealed significant responses in channels 1, 2, 3, 4, 5, 6, 7 and 9 in the LH and channels 13, 14, 15, 16, 18, 19, 20 and 23 in the RH (all p<0.001). In the LH, channels 1, 4, 6, 7 and 9 formed a spatial cluster in which the initial undershoot was significantly different from baseline (p<0.001), and channels 2, 4, 5 and 7 formed a spatial cluster in which the peak of the response was significantly different from baseline (p<0.001). In the RH, channels 18, 20 and 23 formed a spatial cluster with a significant initial undershoot, and channels 13, 14 and 16 formed a spatial cluster with a significant response peak (p<0.001). The permutation test directly comparing the AA and AB conditions showed significantly greater activation to AB than to AA sequences in channels 1, 2, 3, 4, 5, and 7 in the LH and in channels 14, and 17 in the RH (all p<0.001). In the LH, channel 2 formed a spatial cluster in which the initial undershoot was significantly greater (more negative) in the AB than in the AA condition (p<0.001), and channels 1, 4, 5, and 7 formed a spatial cluster in which the peak of the response was significantly higher for AB than for AA (p<0.001). In the RH, channel 14 constituted a spatial cluster with a significant initial undershoot difference, and channel 17 formed a spatial cluster with a significant response peak difference (p<0.001).

*DeoxyHb.* A permutation tests using deoxyHb and comparing the AB condition to baseline showed significant activations (more negative than baseline) in channels 1, 2, 3, 4, 5, 6, 7, 9 and 10 in the LH, and in channels 13-20 in the RH (all p<0.001). Of these, the LH channels 2, 4, 5, 7 and 9 formed a spatial cluster in which the (negative) peak of the response was significantly different from baseline (p<0.001). (No cluster with a significant initial overshoot was found). In the RH, channels 13, 14, 16, 18 and 19 formed a spatial cluster with a significant response peak (p<0.001). The permutation test comparing the AA condition to baseline revealed significant responses in channels 1, 3, 5, 6, 7, and 10 in the LH and channels 13, 14, 15, 16, and 19 in the RH (all p<0.001). In the LH, channels 1, 4, 6, and 7 formed a spatial cluster with a significant peak response (p<0.001). In the RH, channels 13, 14, 16 and 19 formed a spatial cluster with a significant response peak (p<0.001). A permutation test directly comparing the AA and AB conditions showed no significant results.

*Comparing speech, linguistic signs and visual analogs*

An ANOVA directly compared responses to reduplication and control sequences (instead of their difference score, reported in the manuscript) in signs and visual analogs (Experiments 1 and 2 in the current study) and speech (Experiment 1 from Gervain et al. (*5*), testing newborns). The ANOVA was conducted with Stimulus Type (Sign/Visual Analogue/Speech) as a between-subject factor as well as Structure (reduplication/no reduplication) and Hemisphere (LH/RH) as within-subjects factors over oxyHb and deoxyHb concentrations.

For the oxyHb concentrations, we observed a main effect of Structure (F(1,187) = 11.922, p < 0.001) due to higher overall activation to reduplication than to no-reduplication patterns, which is due to the greater activation for the AA patterns in the Sign and Speech conditions. Importantly, we obtained a highly significant Stimulus Type X Structure interaction (F(2,187) = 9.361, p = 0.0001; Figure S1).

Scheffe’s post hoc tests indicated that this interaction is due to the following pairwise comparisons: (a) greater responses to reduplication in the sign and in the speech than in the visual analog conditions (p<0.0001 and p=0.012, respectively); and (b) greater responses to reduplication than to no-reduplication in the sign condition (p<0.0001) as well as in the speech condition (p=0.003).

A similar ANOVA over deoxyHb concentrations yielded a significant main effect of Structure, as reduplication produced overall less negative responses than no-reduplicative sequences (F(1,187)=5.761, p=0.017). Furthermore, the interaction between Stimulus Type and Structure was also significant (F(2,187)=4.64, p=0.010). Scheffe’s post hoc tests indicated that this interaction was due to the stronger (i.e., more negative) response no-reduplicative sequences in the Sign than in the Speech condition (p=0.03).

Figure S1. The oxyHb and deoxyHb concentrations of Experiment 1 and 2, as well as Experiment 1 from Gervain et al. (2008) in the significant clusters

**References**

1. S. Lloyd-Fox *et al.*, Coregistering functional near-infrared spectroscopy with underlying cortical areas in infants. *Neurophotonics* **1**, 025006-025006 (2014).

2. N. Abboub, T. Nazzi, J. Gervain, Prosodic grouping at birth. *Brain and language* **162**, 46-59 (2016).

3. P. T. Fillmore, J. E. Richards, M. C. Phillips-Meek, A. Cryer, M. Stevens, Stereotaxic Magnetic Resonance Imaging Brain Atlases for Infants from 3 to 12 Months. *Developmental Neuroscience* **37**, 515-532 (2015).

4. C. Kabdebon *et al.*, Anatomical correlations of the international 10–20 sensor placement system in infants. *NeuroImage* **99**, 342-356 (2014).

5. J. Gervain, F. Macagno, S. Cogoi, M. Peña, J. Mehler, The neonate brain detects speech structure. *Proc Natl Acad Sci U S A* **105**, 14222-14227 (2008).
